# Impact of Internet Use on Multi-dimensional Health: An Empirical Study Based on CGSS 2017 Data

**DOI:** 10.3389/fpubh.2021.749816

**Published:** 2021-10-18

**Authors:** Junhui Han, Xiaoqiong Zhao

**Affiliations:** School of Economics and Management, Taiyuan University of Technology, Jinzhong, China

**Keywords:** internet use, multi-dimensional health, heterogeneity analysis, mediating effect, endogenous test

## Abstract

Based on the data of the Chinese General Social Survey (CGSS) in 2017, the paper divides overall health into physical, mental, and social health, using the ordered probit model to examine the impact of Internet use on multi-dimensional health. It then discusses the possible heterogeneity in different groups and underlying mechanism. Results found that using the Internet can improve the health level in multiple dimensions. After endogenous and robustness tests, the results remain robust. Heterogeneity analysis shows that Internet use has more obvious effects on the health of senior high school education or above, the elderly, and men. Further analysis of the mediating effect model found that information, leisure, and social preferences are important path mechanisms for Internet use to promote physical, mental, and social health, respectively.

## Introduction

With the implementation of the “broadband China” strategy, China's Internet industry has developed rapidly in recent years. According to the 47th China Statistical Report on Internet Development issued by the China Internet Network Information Center in February 2021, by December 2020, the scale of Chinese netizens has reached 989 million, and the main netizens have begun to transform from youth groups to minors and “silver netizens.” The overall Internet penetration rate has reached 70.4%, the number of mobile Internet users has reached 986 million, and the proportion of netizens using mobile phones to access the Internet is as high as 99.7%. The popularization of the Internet is slowly affecting and changing people's thinking habits and lifestyle. The important manifestation is Internet plus Healthcare. According to the 44th China Statistical Report on Internet Development, as of December 2016, the number of Internet users using Internet medical services has reached 194.76 million, with a utilization rate of 26%.

Health is not only the eternal theme pursued by all mankind but also the cornerstone and destination of high-quality economic and social development. In fact, the Chinese government has constantly attached profound importance to health. The Fifth Plenary Session of the 18th CPC Central Committee clearly proposed to promote the construction of “healthy China;” the “healthy China 2030 plan” officially promulgated by the CPC Central Committee and the State Council pointed out that “people's health should be given priority to the strategic layout of development” in October 2016. The report of the 19th National Congress of the Communist Party of China further emphasizes the significance of the era and strategic significance of the “Healthy China Strategy.” Driven by the dual strategies of “broadband China” and “healthy China,” the Internet is providing more and more people with employment, medical, and other information support, especially the disadvantaged groups. In this context, exploring the relationship between Internet use and residents' health has important policy implications for promoting the development of health.

## Literature Review

### What Is Health?

Health seems to be a mirage. We find difficulty establishing precise definitions when we are in close contact; but when we are far away, we can clearly “see” health (1). Many scholars are concerned with objective health indicators, such as growth and development, prevalence rate, and ADL, among others (2). With the rise of social medicine, subjective health indicators are increasingly proven to be able to successfully predict mortality and disability rate (3). The World Health Organization in 1948 defined health as not only the absence of disease or weakness but also satisfying the need to achieve the perfect state of physical, mental, and social adaptation. Therefore, the complete connotation of health should include multiple dimensions such as physical health, mental health, and good social adaptability (4). However, the current literature mostly focuses on a single dimension of health and lacks a comprehensive investigation of the connotation of health.

### Can Internet Use Affect Individual Health?

At present, two seemingly opposite arguments exist. One is the theory of health promotion which holds that healthy individuals are likely to use the Internet to search for relevant health information (5). In addition, Internet use has a positive role in promoting individual self-reported health (6). By contrast, the theory of technological pressure holds that the Internet popularity has brought negative effects on individual health. Its prominent manifestations are Internet addiction, unreasonable use of the Internet, and excessive dependence on social networking, which will increase the health risk of Internet users (7). How does the Internet affect individual health? First is the mechanism for accessing health information from the Internet. Access to health information through the Internet and online health services can improve individual health literacy and strengthen self-health management (8). The Internet has been said to have become an effective means to obtain health information and prevent various diseases (9, 10). Second is the interpersonal emotional interpretation mechanism of the Internet (11). The mechanism accepts as true that the Internet, as a medium of social communication, can alleviate users' loneliness and anxiety and then improve their health (12). Social networking can also relieve pain and reduce the probability of depression (13, 14). Apart from physical and mental health, the Internet can also improve residents' enthusiasm to participate in community activities (15). This idea means that Internet use can have a positive impact on individual social adaptability or social health, but only a small amount of literature has explored this assumption.

### Which Groups Are More Affected by Internet Use?

From the perspective of age, adolescents, and the elderly are the focus of literature. The study found that approximately 14.5% of Chinese young netizens have ever caused property loss or physical and psychological injury due to false information on the Internet; some young people have also suffered varying degrees of personal and behavioral injuries due to excessive addiction to online games. Internet use time affects the health status of adolescents by changing their life time allocation (16). For example, with the increase in online time, teenagers' sleep and exercise time are often squeezed out (17). In addition, the Internet has begun to play an increasingly important role in the lives of the elderly. People aged 60–70 with higher socioeconomic status are more likely to use the Internet (18, 19). The elderly keep close contact with their children, relatives, and friends mainly through e-mail and other forms (20). In general, Internet use not only can improve the physical health of the elderly but also increase their subjective well-being (21, 22).

### A Brief Review

To sum up, from the perspective of academic research, it still requires improvement from the following aspects. First of all, the literature mostly focuses on a single dimension of health and lacks multi-dimensional consideration of health connotation. Second, given the possible reciprocal cause–effects between Internet use and individual health, we should further consider the endogenous problem. Regular or moderate Internet use can improve health. For example, it can relax oneself, relieve anxiety and so on. But excessive addiction to the Internet will lead to decline in health. In reverse, anxiety, loneliness, and other factors can also affect Internet use, especially the Internet addiction. That is to say, there is a causal endogeneity between them. Therefore, an instrumental variable model is necessary for correction. Third, the formation mechanism of social health may be ignored due to the single research dimension.

The purpose of this paper is to explore the effects of Internet use on Multi-dimensional health. First, the study divides the overall self-reported health into three dimensions: physical, mental, and social health, and then examines the impact of Internet use on health in each dimension. Second, Eoprobit model is used to test endogeneity, and substitution variables, linear regression model, and Heckman model for the robustness test. Third, considering the heterogeneity of the sample, we use the interaction term model to test the impact of Internet use on the health of different groups. Fourth is through the mediating effect model to explore the underlying mechanism of Internet use affecting physical, mental, and social health.

## Data Sources and Econometric Models

### Data Sources

The data used in this paper are from the CGSS. This database was completed by the Department of Social Sciences, Renmin University of China in cooperation with the Survey Research Center of the Hong Kong University of Science and Technology. Five-year (2003–2008, excluding 2007) high-quality survey data have been completed in the first phase. Then, seven surveys were conducted in the second phase (2010–2019, excluding 2014). This survey item mainly focuses on the major theoretical and practical problems in the change of China's social structure and comprehensively collects some basic information about residents' behavior mode, thinking mode, lifestyle, and social change. At present, this database has become an important reference for academic research and policy formulation. The CGSS questionnaire includes questions on individual health and Internet use. According to the respondents' answers, we can measure these variables. Considering the timeliness and availability of the data, this paper uses the 2017 CGSS data. Following the purpose of the research, 9,808 samples were retained by deleting missing values and outliers.

### Variable Selection

#### Dependent Variable

According to the ideas of Rosini (4) and Wang (23), the paper uses the self-reported health indicator to measure general health; the items of “health affects the frequency of work and life,” “the frequency of depression or depressed,” and “the frequency of participating in social activities or visiting in free time” in the questionnaire are, respectively, used to measure physical, mental, and social health. According to the respondents' answers, health status is divided into five levels with values ranging from 1 to 5. The higher the value, the higher the level of health.

#### Independent Variable

The independent variable is Internet use. According to the respondents' answers in the 2017 CGSS, we assign 1 to “never,” 2 to “rarely,” 3 to “sometimes,” 4 to “often,” and 5 to “always.”

#### Control Variables

Including two types of control variables at the individual and family levels. The former includes gender, age, marital cohabitation, party member status, education, and physical exercise. The latter includes family economic status and household register. Considering that anxiety and other health problems are often closely related to emotional and financial support of family members, especially spouses (couples). Therefore, we design the variables of marital cohabitation according to whether they live together or not, which is different from most previous literature. We assigned 0 to the four situations of “unmarried,” “separated without divorce,” “divorced,” and “widowed” in the questionnaire which are regarded as “divorced;” and 1 to other situations, which means “married.”

#### Mediating Variables

This paper argues that Internet users have different preferences. For example, some people prefer watching movies on the Internet, while others may prefer to search for relevant information. According to the three dimensions of physical, mental, and social health, this paper proposes three corresponding mediating variables: information, leisure, and social preferences. Information preference is expressed by “the frequency of online access to information in the past year,” leisure preference is expressed by “the frequency of online participation in leisure entertainment (games, music, video, etc.) in the past year,” and social preference is expressed by “the frequency of online participation in social activities in the past year.” The three variables are all five-level sequence category variables. 1 means “never,” 2 means “little,” 3 means “sometimes,” 4 means “often,” and 5 means “always.” Table 1. shows the specific variable settings.

**Table 1:** Descriptive statistics of samples.

**Variables**	**Mean value**	**Standard deviation**	**Minimum value**	**Maximum value**	**Explain**
Physical health	4.027	1.072	1	5	Ordered classification variables, assigned to 1–5
Mental health	3.809	0.990	1	5	Ordered classification variables, assigned to 1–5
Social health	2.745	1.022	1	5	Ordered classification variables, assigned to 1–5
General health	3.6037	1.073	1	5	Ordered classification variables, assigned to 1–5
Internet use	3.173	1.679	1	5	Always = 5, often = 4, sometimes = 3, rarely = 2, never = 1
Gender	0.467	0.499	0	1	Male = 1, female = 0
Age	44.813	13.180	18	65	Survey year minus birth year
Marital cohabitation	0.800	0.400	0	1	Unmarried, separated without divorce, divorced, and widowed = 0, others = 1
Party member status	0.093	0.290	0	1	Party membership = 1, others = 0
Education	9.705	4.093	1	15	No formal education = 1, primary school = 6, junior high school = 9, senior high school = 12, junior college or above = 15
Physical exercise	2.505	1.555	1	5	The frequency of participating in physical exercise is 1–5
Household register	0.361	0.480	0	1	Agricultural household registration = 0, Non-agricultural household registration = 1
Family economic status	2.564	0.738	1	5	Family economic status level, assigned to 1–5
Information preference	3.550	1.069	1	5	Five level sequence category variable
Leisure preference	3.349	1.107	1	5	Five level sequence category variable
Social preference	3.705	1.074	1	5	Five level sequence category variable

### Empirical Model

Two types of empirical models are involved in this paper. One is the regression of ordered probit model on health, and the other refers to the three mediating effect models based on information, leisure, and social preferences.

First, we set up the following ordered probit model about health for ordered categorical variables. Assuming that the healthy value range is 1, 2,., *m*, then, the ordered probit model can be set as follows:

We assume that *Y*_*i*_ = *j*.when we satisfy


(1) $$\begin{array}{l} u_{j-1}<{Y_{i}}^{*}\leq u_{j},j=1,2,...,m. \end{array}$$

$Y_{i}^{*}$ is the latent variable of the ordered categorical variable $Y_{i}^{\;}$ and is affected by the Internet use *Inter*_*i*_ and the control variable *X*_*i*_. It can be expressed as follows:


(2) $$\begin{array}{l} {Y_{i}}^{*}=\beta Inter_{i}+\gamma X_{i}+u_{i}. \end{array}$$

When we satisfy*u*_*j*_ ≤ *u*_*j*+1_, *u*_0_ = −∞, *u*_*m*_ = +∞, the probability of*Y*_*i*_ = *j* can be expressed as follows:


(3) $$\begin{array}{l} pr\left(Y_{i}=j\right)=\Phi \left(u_{j}-\beta Inter_{i}-\gamma X_{i}\right)\\ \;-\Phi \left(u_{j-1}-\beta Inter_{i}-\gamma X_{i}\right). \end{array}$$

Φmeans the cumulative density function that obeys the standard normal distribution and satisfies *j* = 1, ..., 5.

Second, according to information, leisure, and social preferences, we set up the following three mediating effect models.

According to the mediating effect test procedure proposed by Wen et al. (24), this paper uses the following Equations (4)–(6). For convenience, the control variables are omitted here, and only social preference is used as an example.


(4) $$\begin{array}{l} Y_{i}=c\times Inter_{i}+\varepsilon_{i}, \end{array}$$

(5) $$\begin{array}{l} socialprefer_{i}=a\times Inter_{i}+\mu_{i}, \end{array}$$

(6) $$\begin{array}{l} Y_{i}=c^{{\;}^{\prime }}\times Inter_{i}+b\times socialprefer_{i}+\nu_{i}. \end{array}$$

In Equation (4), *c* is the total effect of the independent variable *Inter*_*i*_ (Internet use) on the dependent variables *Y*_*i*_(health); in Equation (5), *a* is the effect of the independent variable *Inter*_*i*_(Internet use) on mediating variables Socialprefer (Social preference); in Equation (6), *b* is the effect of mediating variable Socialprefer (Social preference) on dependent variable *Y*_*i*_ (health), and *c*′ is the direct effect of the independent variable*Inter*_*i*_ (Internet use) on the dependent variable (health) after controlling Social preference. Following the idea of Wen et al. (24), we first test whether the regression coefficient *c* is significant. If significant, it should be judged according to the mediating effect; otherwise, it should be judged according to the masking effect. Second, we judge the significance of regression coefficients *a* and *b*; if they are both significant, the indirect effect is significant. We then further test the significance of the direct effect *c*′ of Social preference on *Y*_*i*_ (health). If the coefficient *c*′ is significant, a partial mediating effect possibly exists. Otherwise, a complete mediating effect is considered to exist.

The above basic model may evidently cause inconsistent and biased estimates due to the sample self-selection bias or mutual causality between Internet use and individual health. Later, We will use the Heckman model and Eoprobit model to solve these problems.

## Empirical Results

### Impact of Internet Use on Individual Multi-Dimensional Health

The general, physical, mental, and social health are divided into five ordered levels, i.e., 1, 2, 3, 4, 5, while controlling many variables such as individuals and family characteristic and using the regression of ordered probit model to investigate the impact of Internet use on individual's multi-dimensional health. Table 2 shows the results.

**Table 2:** Ordered probit estimation of Internet use on individual multidimensional health.

**Variables**	**General health**	**Physical health**	**Mental health**	**Social health**
Internet use	0.0752*** (0.00928)	0.0949*** (0.00964)	0.0403*** (0.00937)	0.0392*** (0.00917)
Gender	0.0833*** (0.0220)	0.0738*** (0.0229)	0.0931*** (0.0221)	−0.110*** (0.0216)
Age	−0.0251*** (0.00115)	−0.0135*** (0.00119)	−0.00108 (0.00114)	−0.00157 (0.00112)
Marital cohabitation	0.137*** (0.0293)	0.155*** (0.0304)	0.217*** (0.0291)	0.0915*** (0.0286)
Party member status	0.109*** (0.0395)	0.117*** (0.0423)	0.112*** (0.0401)	0.0505 (0.0385)
Education	0.0281*** (0.00379)	0.0372*** (0.00390)	0.0265*** (0.00379)	−0.00805** (0.00373)
Physical exercise	0.0747*** (0.00757)	0.0593*** (0.00789)	0.0666*** (0.00763)	0.0616*** (0.00745)
Household register	−0.0356 (0.0265)	0.0660** (0.0278)	0.0845*** (0.0266)	−0.105*** (0.0260)
Family economic status	0.00296** (0.00148)	0.00505*** (0.00166)	0.00422*** (0.00153)	0.000347 (0.00144)
Pseudo-*R*^2^	0.0737	0.0606	0.0224	0.0508
Observations	9,808	9,808	9,808	9,808

The parentheses are standard errors. ***, **, and * indicate significance at 1, 5, and 10% levels, respectively. Owing to space limitation, the estimation of cut-off points is omitted here.

From the control variables, marital cohabitation, age, gender, and household register have different directions and degrees of influence on individual multi-dimensional health. Compared with women, men's general, physical, and mental health levels are higher, but women's individual social health level is higher than men's. Overall, the regression coefficients of gender are significant at the level of 0.01. With the growth of age, the health level of each dimension begins to decline, but age had no significant effect on the latter two types of health. The regression coefficients of married cohabitation are all positive in the four regression equations and are significant at the level of 0.01, implying that cohabitation can improve the general health and the health levels of all dimensions. In fact, living together with a spouse or couple can provide emotional or financial support for each other, which will somewhat reduce anxiety and relieve stress. Party member status only has a certain positive impact on the general, physical, and mental health. The higher the education, the higher the level of general, physical, and mental health. However, a negative relationship exists between the education and social health levels, which may mean that the higher the education level, the lower the possibility of participating in social activities and visiting. In addition, the frequency of participating in physical exercise is positively correlated with the level of health in all dimensions. At the same time, the higher the family's economic status, the higher the health level except social health. Non-agricultural household registration individuals show higher physical and mental health levels. Meanwhile, agricultural household registration has a higher social health level, showing certain “optimistic” characteristics.

Table 2 also shows that the regression coefficients of Internet use on general health, physical health, mental health, and social health are all positive and significant at the level of 0.01, which means that Internet use can improve the health level in multiple dimensions. Because multi-dimensional Health are ordinal categorical variables, the regression coefficients in Table 2 only reflect their effective degree on health, not marginal effects. Therefore, the marginal effect of Internet use on multi-dimensional health was investigated based on the estimates of each cut-off points. Table 3 shows the results. Taking general health as an example, when Internet use increases by one unit, the probability of overall health status as “very unhealthy,” “relatively unhealthy,” and “average” will decrease by 0.51, 1.13, and 0.94%, respectively. The probability of being “healthier” and “very healthy” increased by 0.63 and 1.96%. Similar explanations can be made for physical, mental, and social health.

**Table 3:** The marginal effect of oprobit model.

**Variables**	***Y* = 1**	***Y* = 2**	***Y* = 3**	***Y* = 4**	***Y* = 5**
**Internet use**	**General health**				
	−0.0051***	−0.0113***	−0.0094***	0.0063***	0.0196***
	(0.0006)	(0.0014)	(0.0011)	(0.0007)	(0.0024)
	**Physical health**				
	−0.0054***	−0.0103***	−0.0120***	−0.0060***	0.0338***
	(0.0006)	(0.0010)	(0.0012)	(0.0006)	(0.0034)
	**Mental health**				
	−0.0016***	−0.0051***	−0.0079***	0.0014***	0.0132***
	(0.0003)	(0.0012)	(0.0018)	(0.0003)	(0.0030)
	**Social health**				
	−0.0071***	−0.0082***	0.0031***	0.0087***	0.0034***
	(0.0016)	(0.0019)	(0.0007)	(0.0020)	(0.0008)

Delta-method standard error in parentheses. ***, **, and * indicate significance at 1, 5, and 10% levels, respectively.

**Figure 1:**
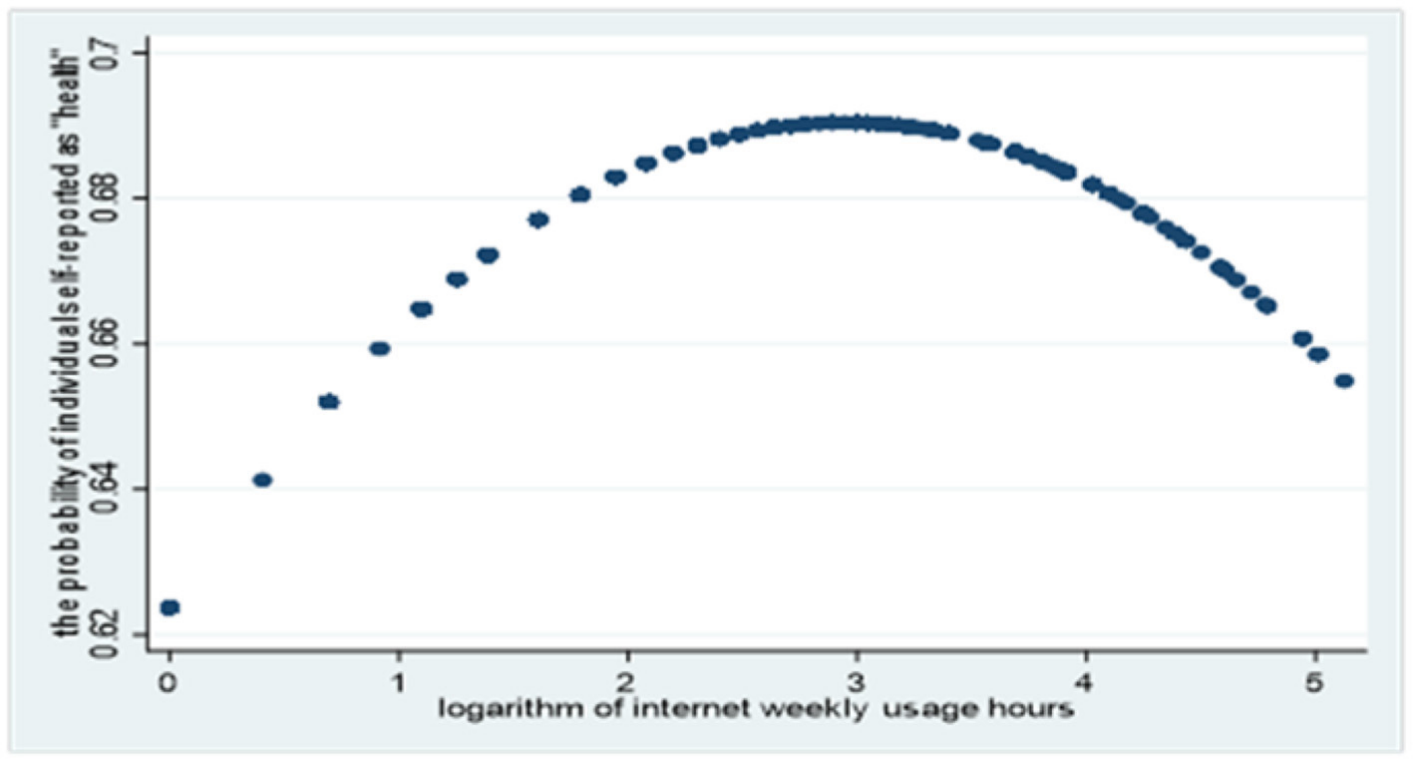
Relationship between the logarithm of Internet weekly usage hours and the degree of depression.

As the research literature points out, excessive use of the Internet may have a negative impact on health. This paper takes the mental health dimension as an example to discuss the relationship between the time spent on the Internet and the “frequency of individual's depression or depressed.” We consider “never” and “little” as “healthy” and assign them to 1, but we deem other cases “unhealthy” and thus assign them to 0. By estimating the predicted probability that the mental health level is “healthy,” a significant inverse U relationship is found between the predicted probability value and the logarithm of the number of online hours per week as shown in Figure 1. With the increase in Internet use time, the degree of individual psychological depression will be significantly reduced or the probability of individual self-reported as “health” will increase. However, when the logarithm of the number of online hours per week exceeds a certain threshold, it will have a negative impact on individual's mental health, and the probability of individual self-reported as “health” will continue to decline.

### Endogenous Test

The impact of Internet use on health status is investigated above, but other influencing factors may be present because of the availability of data. At the same time, Individual with good health status may have more energy and time to surf the Internet. Therefore, this study may face an endogeneity problem caused by an omitted variable and reciprocal cause–effects relationship. The instrumental variable is an effective method for endogeneity problems. Theoretically, effective instrumental variables must be uncorrelated with random disturbances. Meanwhile, they must be highly correlated with endogenous variables. According to the CGSS 2017 data, we use other family members' Internet access in the past 6 months, referred to as Family internet use as an instrumental variable. Firstly, Family internet use is closely related to individual Internet use. After all, online communication is an important way for family members to maintain affection in the Internet age. Secondly, the health status of an individual depends mainly on self-responsible factors, and has little to do with the frequency of Internet access of other family members. Specifically, this variable is assigned to 1 when other family members have used Internet in the last 6 months, and 0 otherwise.

Since Internet use is a discrete variable, the traditional instrumental variable model cannot solve this problem. For this reason, the paper uses the Eoprobit regression model in extended regression models (ERMs) to solve the endogeneity problem when the independent variable is discrete variable in ordered probit model. Limited by space, here only lists the test results of the general health. Table 4 shows the results.

**Table 4:** Results of endogenous test.

**Variables**	**General health**	**Internet use**
Gender	0.0845***	0.0147
	(0.0221)	(0.0235)
Age	−0.0143***	−0.0526***
	(0.00240)	(0.00108)
Marital cohabitation	0.0750**	0.209***
	(0.0318)	(0.0314)
Party member status	0.0628	0.262***
	(0.0409)	(0.0415)
Education	0.00326	0.126***
	(0.00620)	(0.00379)
Household register	−0.120***	0.444***
	(0.0306)	(0.0276)
Physical exercise	0.0693***	
	(0.00752)	
Family economic status	0.00254*	
	(0.00146)	
Internet use	0.246***	
	(0.0335)	
Family internet use		0.859***
		(0.0327)
Observations	9,683	9,683
Corr (e.general health, e.internet use)	−0.2082***	
	(0.0396)	

The parentheses are standard errors. ***, **, and * indicate significance at 1, 5, and 10% levels, respectively. Owing to space limitation, the estimation of cut-off points is omitted here.

On the one hand, the regression coefficient of Internet use to general health is 0.246 and significant at the level of 0.01 in the main regression equation. On the other hand, the regression coefficient of Family internet use to Internet use is 0.859 in the auxiliary regression equation, which is significant at the level of 0.01. The correlation coefficient of the residuals of the two equations is −0.2082 and significant at 0.01 level, which means Internet use is an endogenous variable and the unobservable factors that affect Internet use reduce an individual's health level. But why? As shown in Figure 1, excessive addiction to the Internet will lead to decline in health. Some unobservable factors such as doldrums and loneliness related to Internet addiction may have greater negative effects on health. So, the negative sign maybe expected. The above results indicate that Internet use is an endogenous variable. Family internet use has strong explanatory power to Internet use. The findings that Internet use improves general health status remained robust after accounting for endogeneity.

### Robust Test

In this section, substitution variables, linear regression model, and Heckman model were used to test the robustness. Again, take general health as an example. In the first method, Frequency of surfing the Internet in free time is used as a substitute variable for Internet use. According to the item in the CGSS data in 2017, “never,” “Several times a year or less,” “Several times a month,” “Several times a week,” and “Every day” were assigned a value of 1–5, respectively. It can be seen from the first column of Table 5 that frequency of surfing the Internet in free time has a significant positive impact on general health. If we use linear regression instead of ordered probit model, Internet use remains a significant impact on overall health. In the Heckman two-step selection model, the first stage is to establish the Internet use decision equation, the outcome equation in the second stage examines the effect of Internet use on general health. The results of selection equation are not presented in the Table 5 due to lack of space. As shown in the third column, rho = −0.1458 and LR test = 10.05, indicating that the null hypothesis that the correlation coefficient between selection and outcome equation is equal to 0 should be rejected. Thus, the two equations are related. There will be sample selection bias if we don't estimate two equations simultaneously, so the Heckman model is effective and necessary. Based on the selection equation, the regression coefficient of the Internet use is 0.0575, which means that as the frequency of Internet use increases, so does the general health level.

**Table 5:** Results of robust test.

**Variables**	**Substitution variable**	**Linear regression**	**Heckman model**
Internet use		0.0730***	0.0575***
		(0.0083)	(0.0127)
Frequency of surfing the Internet in free time	0.0690***		
	(0.0082)		
*R*^2^/Pseudo-*R*^2^	0.0739	0.1950	
Rho			−0.1458***
			(0.0462)
LR test			10.05***

The parentheses are standard errors. ***, **, and * indicate significance at 1, 5, and 10% levels, respectively. Owing to space limitation, the estimation of cut-off points is omitted here.

### Heterogeneity Analysis

As emphasized in the research literature, Internet use may have different effects on adolescents and the elderly, which means there may be heterogeneity in the health effects of Internet use. This section only takes the general health as an example and adds the interaction items of Internet use and education, age, and gender into the basic model to study the heterogeneity of Internet use affecting general health. For comparison between groups, education and age are treated as binary dummy variables. That is, education is assigned to 1 when the level of education is higher than high school, otherwise, it is 0; Age is assigned to 1 when individual biological age is between 36 and 65 years old, it is 0 when individual biological age is between 18 and 35 years old. When the ordinal regression model incorrectly assumes that error variances are the same for all groups in the population, the parameter estimates will be biased. Different age, education and gender groups may have different health outcomes. So, we use the heteroskedastic ordered models proposed by Williams (25) for research. This models simultaneously fit two equations, one for the means model and one for the residual variance, thereby allowing the variance to differ across all groups in the population. Table 6 shows the results.

**Table 6:** Heterogeneity of Internet use affecting general health.

	**Control variable**	**Controlled**	**Controlled**	**Controlled**
**Means model**	Internet use × education	0.157***		
		(0.0267)		
	Internet use × age		0.0066***	
			(0.000790)	
	Internet use × gender			0.171***
				(0.0281)
**Variance model**	Education	−0.163***		
		(0.0194)		
	Age		0.00487***	
			(0.000781)	
	Gender			0.0541***
				(0.0183)
	Pseudo-*R*^2^	0.0712	0.0454	0.0725
	LR test	69.26***	38.52***	8.75***
	Observations	9808	9808	9808

The parentheses are standard errors. ***, **, and * indicate significance at 1, 5, and 10% levels, respectively. Owing to space limitation, the estimation of cut-off points is omitted here.

As seen from the upper part of the Table 6, also controlling the relevant variables, the interaction items between Internet use and education, age, and gender are 0.157, 0.0066, and 0.171, respectively, and they are significant at the 0.01 level. The lower part of the Table 6 shows these variables proved to be statistically significant determinants of the residual variance—e.g., the level of education is higher than high school (reduced variance), biological age is between 36 and 65 years old (increased variance), and being male (increased variance)—these include our variable of interest. A certain degree of heterogeneity is indicated to exist in the general health effects of Internet use on individuals with different characteristics. Specifically, the effect of Internet use on health promotion of high school or above education group is significantly greater than that of groups with a degree below high school, and Internet use has a greater role in promoting the general health level of the elderly and male groups.

### Mediating Effect of Internet Use Preference

The above results show that Internet use has a significant promoting effect on each dimension of health, and heterogeneity exists among different groups. Furthermore, how does Internet use affect health in all dimensions? In fact, different individuals have different Internet usage preferences, which may lead to different health outcomes. Combined with the CGSS data in 2017, this paper argues that Internet use preference is an important path mechanism for Internet use to promote health. It also attempts to test the mediating effect of Internet use preference on the relationship between Internet use and health. For physical health, the Internet is an important health information dissemination channel, which can improve the personal health literacy and quality of life. Therefore, the paper uses Equations (4)–(6) to test the mediating effect of information preference between Internet use and physical health. Similarly, we examine the mediating effects of leisure and social preferences between Internet use and mental or social health.

Table 7 reports the mediating effects of information, leisure, and social preferences in three parts. The results of the first part show that the regression coefficient of Internet use to information preference is 0.3462, and it is significant at the level of 0.01. This finding indicates that the two are highly correlated. Furthermore, when Internet use and information preference are simultaneously included in the regression equation, the direct impact of Internet use on physical health is found to be 0.0420 and significant at the level of 0.1. Meanwhile, the regression coefficient of information preference is 0.0880 and significant at the level of 0.01. Combining the results of the total effect of Internet use on physical health in the second column of Table 2, information preference is considered to play a partial mediating role in the relationship between Internet use and physical health. The mediating effects of leisure and social preferences can undergo similar analysis, which will not be repeated here.

**Table 7:** Test results of mediating effect.

**Variables**	**Dependent variable: information preference**	**Dependent variable: physical health**
**Mediating effect of information preference**		
Information preference		0.0880***
		(0. 0258)
Internet use	0.3462***	0.0420*
	(0.0244)	(0. 0262)
Other variables	controlled	controlled
Observations	2247	2247
Pseudo-*R*^2^	0.0997	0.0324
**Mediating effect of leisure preference**		
**Main variables**	**Dependent variable: leisure preference**	**Dependent variable: mental health**
Leisure preference		0.0386*
		(0. 0221)
Internet use	0.2447***	0.0526**
	(0.0241)	(0.0237)
Other variables	controlled	controlled
Observations	2247	2247
Pseudo-*R*^2^	0.0629	0.0072
Mediating effect of social preference		
**Main variables**	**Dependent variable: social preference**	**Dependent variable: social health**
Social preferences		0.1107 ***
		(0.0233)
Internet use	0.3458***	0.0520 **
	(0.0243)	(0. 0247)
Other variables	Controlled	Controlled
Observations	2,247	2,247
Pseudo-*R*^2^	0.0770	0.0129

The parentheses are standard errors. ***, **, and * indicate significance at 1, 5, and 10% levels, respectively. Owing to space limitation, the estimation of cut-off points is omitted here.

## Conclusion and Enlightenment

On the bases of the data of the 2017 CGSS, this paper examines the impact of Internet use on general, physical, mental, and social health. It then explores the heterogeneity of the impact among different groups and its underlying mechanism. The results show that Internet use has a significant positive impact on all dimensions of health. After endogenous and robustness tests, the results confirm the robustness of the conclusion that Internet use can improve health level.

Through the analysis of the possible heterogeneity in different groups, the results show that the impact of Internet use on individual health varies significantly among different educational background, age, and gender. Internet use has more obvious health effects on senior high school education, the elderly, and the males. Furthermore, the analysis of the mediating effect model identifies information, leisure, and social preferences as the important path mechanisms for Internet use to promote physical, mental, and social health.

In view of the above conclusions, this paper puts forward the following policy recommendations. First is the need to further improve Internet penetration. Although the overall Internet penetration rate in China has reached 70.4% by December 2020, differences persist between urban and rural areas and regions. According to the 2017 CGSS data, among the 1,789 individuals who did not surf the Internet, 32.03% did not know how to surf the Internet, 2.96% had no equipment or place to surf the Internet, and 26.33% could not surf the Internet. Therefore, we should further strengthen the construction of Internet infrastructure, increase the access opportunities of the entire society, and lay the foundation for improving the health effect of the Internet. Second is to provide differentiated and high-quality Internet services. In view of the heterogeneity of Internet health effects, the government should establish health information platform for adolescents, middle-aged, and elderly people and implement “Internet plus precision health” to provide differentiated high-quality health information for different social groups. At the same time, we should further enrich the content of Internet services, appropriately increase Internet entertainment and leisure functions, and then meet diversified needs. Third is to cultivate a good network culture and enhance the comprehensive scientific literacy of netizens. In the Internet age, all kinds of “fresh” information emerge endlessly. Different Internet usage preferences may lead to different health outcomes. Therefore, we should cultivate a healthy, green, civilized, and harmonious Internet culture in the entire society to promote individuals' formation of a good healthy choice mechanism. We must also enhance the individual's comprehensive ability to “access the Internet” and minimize the negative effects of Internet use.

## Data Availability Statement

Publicly available datasets were analyzed in this study. This date can be found here: http://cgss.ruc.edu.cn.

## Author Contributions

JH: research conceptualization, methodology, validation, resources, data curation, writing—original draft preparation, supervision, project administration, and funding acquisition. XZ: software, formal analysis, investigation, writing—review and editing, and visualization. All authors have read and agreed to the published version of the manuscript.

## Funding

We gratefully acknowledge the financial support from the National Social Science Fund of China (Grant No. 20BSH128).

## Conflict of Interest

The authors declare that the research was conducted in the absence of any commercial or financial relationships that could be construed as a potential conflict of interest.

## Publisher's Note

All claims expressed in this article are solely those of the authors and do not necessarily represent those of their affiliated organizations, or those of the publisher, the editors and the reviewers. Any product that may be evaluated in this article, or claim that may be made by its manufacturer, is not guaranteed or endorsed by the publisher.
